# Living is very dangerous: dialysis in the pandemic

**DOI:** 10.1590/2175-8239-JBN-2022-E008en

**Published:** 2022-11-28

**Authors:** Dirceu Reis da Silva

**Affiliations:** 1 Hospital de Clínicas de Porto Alegre, Porto Alegre, RS, Brazil; 2 Instituto de Doenças Renais, Porto Alegre, RS, Brazil

Since the beginning of the covid-19 pandemic, we have known that the chronic kidney population has a greater chance of contamination and disproportionate illness development^1^, which can be significantly impacted in the contexts that characterize each treatment modality. Since the beginning of the pandemic, expectations have grown that peritoneal dialysis would offer a lower risk of contamination due to reduced exposure to clusters of patients and healthcare professionals and environments, with the online availability of care and prescription adjustments, in addition to not depending on healthcare workers to carry it out at home (often contaminated and away from work teams, with consequent difficulty in maintaining activities)^2^,^3^. In addition, hemodialysis involves greater complexity in the supply of machinery, treated water and supplies, due to the overload of the hospital network, that sought to serve the overcrowded intensive care units^2^.

The study by Gorayeb-Polacchini et al.^4^ helps us understand how the pandemic impacted patients in a Brazilian dialysis program. Essentially, hemodialysis patients had a higher incidence of infections by covid-19, but those on peritoneal dialysis developed more severe conditions, with greater need for hospitalization, ventilatory support, intensive care, and higher lethality; the authors then suggest parsimony in the indication of changing the modality (from hemodialysis to peritoneal dialysis) aiming to protect chronic kidney patients, suggested at the beginning of the pandemic^5^. Both in the general population and in the chronic kidney segment, the health problems caused by the covid-19 pandemic were being impacted by the adoption of social distancing measures, the offer of testing, the use of vaccines, the emergence of viral variants, and the recurrent involvement of individuals who may have already been exposed. With regards to vaccines, we need to consider the greater difficulty in achieving a consistent vaccine response^6^, the categories of vaccines and the availability or not of consistent data on safety and efficacy in chronic kidney disease. Vaccines available in Brazil efficiently generate antibody titers in chronic kidney patients, whether they are attenuated virus vaccines, non-replicating viral vector vaccines, or those based on messenger RNA technology^7^. In particular, there seems to be a more preserved vaccine response in chronic renal patients on peritoneal dialysis than in those on hemodialysis^7^, presumably due to a less pronounced inflammatory status and better preservation of residual renal function with optimized removal of higher molecular weight uremic toxins. In addition, the application of a booster vaccine dose (mRNA) in patients on peritoneal dialysis leads to response optimization in most patients^8^. Due to the fact that the vaccine schedules were not yet fully applied in the chronic kidney population, the higher mortality in peritoneal dialysis reported by Gorayeb-Polacchini et al.^4^ may reflect the failure of the vaccine status at the time.

It is also important to pay attention to the need to offer different treatment alternatives, and to size the adequate implementation of the protective measures indicated in the pandemic. Nordio et al.^9^ describes a survey by the Italian Society of Nephrology regarding fixed and contextual factors, noting that, in patients undergoing hemodialysis, there was a direct relationship between contamination by covid-19 and infection of the healthcare staff, the policy of extensive testing and incidence in the general population; in addition, the duration of the lockdown exerted a protective effect. The predictive factors of infection in the population on peritoneal dialysis were the location of the center and the proportion of infected people in the general population. These findings suggest that different intensities of these factors can impact different perceptions of risk associated with dialysis modalities. In Argentina, despite greater contamination in hemodialysis patients, mortality was the same in both treatment options^10^. As an example, extensive testing of the population was deficient in Brazil until at least the second half of 2020 due to international commercial competition for tests, problems in the capacity of the collection and processing network, and the adoption of non-standardized national and regional policies^11^.

Considerations such as better quality of life, preservation of residual renal function (an important predictor of survival) and cost-effectiveness should naturally be analyzed in the recommendations relevant to the choice of dialysis modality. With regards to our pandemic times, the full application of good practices of social distancing, the use of masks, testing, the use of antiviral treatments, and even specific prophylaxis in immunosuppressed people, is essential for the best framing of the benefits and risks associated with each alternative. The recommendations relevant to care in dialysis programs are added, with peritoneal dialysis being included here, with the aim of maximum protection for patients^5^,^12^.

We are still learning about the impact of the pandemic on chronic kidney disease and about pragmatic protection strategies. The data from the study by Gorayeb-Polacchini et al.^4^ are very welcome in such an unexplored scenario as the one we face, and gives us an essential alert, to be kept alive in memory, meaning the balance between widespread vaccination prevails and the expressive gain of expertise of care organizations.
